# The Multifaceted Role of Extracellular Vesicles in Alzheimer's Disease

**DOI:** 10.1111/jnc.70209

**Published:** 2025-08-27

**Authors:** Anna R. R. Da Conceicao, Júlia Marinatto, Lisandra S. Pinheiro, Tayna Rody, Fernanda G. De Felice

**Affiliations:** ^1^ Institute of Medical Biochemistry Leopoldo de Meis Federal University of Rio de Janeiro Rio de Janeiro Brazil; ^2^ D'Or Institute for Research and Education Rio de Janeiro Brazil; ^3^ Centre for Neuroscience Studies Queen's University Kingston Ontario Canada; ^4^ Department of Biomedical and Molecular Sciences Queen's University Kingston Ontario Canada; ^5^ Department of Psychiatry Queen's University Kingston Ontario Canada

**Keywords:** Alzheimer's disease, biomarkers, extracellular vesicles, neuroinflammation, neuroprotection

## Abstract

Extracellular vesicles (EVs) are lipid bilayer nano‐ to micro‐sized particles that carry biomolecules, such as proteins, lipids, and genetic material. Their composition depends on the cellular microenvironment and the health status of tissues. EVs are released by different cell types under distinct circumstances, mediating intercellular communication in both physiological and pathological contexts. In Alzheimer's disease (AD), EVs have been shown to influence pathological events, carrying neurotoxins, such as neuroinflammatory factors, pathogenic forms of amyloid‐β, and phosphorylated tau into recipient neurons. This contributes to the propagation of AD pathology and exacerbates neuronal degeneration. However, under physiological conditions, EVs play key roles in maintaining tissue homeostasis. In the central nervous system (CNS), EVs derived from glial cells and neurons modulate synaptic plasticity and neuronal activity. Interestingly, EVs carrying neurotoxin molecules can cross the blood–brain barrier, making them attractive candidates as biomarkers for diagnosis with a minimally invasive approach to assess CNS alterations. Additionally, EVs contribute to the activation of neuroprotective pathways, participating in the periphery‐to‐brain signaling. Notably, alteration of EV content has been further proposed to have potential therapeutic applications. Herein, we summarize the multifaceted role of EVs in AD, emphasizing their role in promoting neuroprotection and exploring their contribution to our understanding of AD pathophysiology.

## Graphical abstract

Extracellular vesicles (EVs) exhibit diverse functions depending on the context of their release, and they can be divided into physiological, Alzheimer's disease, biomarkers, and therapeutic. (A) EVs mediate intercellular communication by transporting key trophic factors, such as brain‐derived neurotrophic factor (BDNF); they also support homeostatic maintenance through the release of anti‐inflammatory cytokines, microRNAs (miRNAs), and small interfering RNAs (siRNAs) and by sequestering misfolded proteins for lysosomal degradation. (B) EVs can carry pro‐inflammatory cytokines and facilitate the spread of neurotoxic species in Alzheimer's disease, including amyloid‐β oligomers (Aβ) and phosphorylated tau (p‐Tau). (C) EVs can be used as minimally invasive biomarkers for disease diagnosis and monitoring, particularly when assessed alongside established clinical parameters. (D) EVs attract interest as therapeutic vehicles, as their cargo can be engineered to deliver neuroprotective agents, such as anti‐inflammatory compounds, exerkines, miRNAs, and siRNAs, thereby enhancing brain resilience and improving neuronal health. The figure was created with BioRender.com.

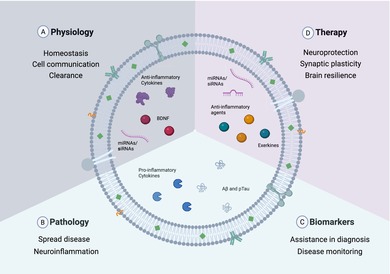

**Abbreviations**
AD: Alzheimer's disease
ADEVs: astrocyte‐derived EVs
ADMSC: adipose‐derived mesenchymal stem cells
AdSCs: adipose tissue‐derived stem cells
ALIX: ALG‐2 interacting protein X
APOE: apolipoprotein E
APP: amyloid‐beta precursor protein
APP‐CTFs: amyloid precursor protein‐derived C‐terminal fragments
ATN: amyloid deposition (A), tau pathology (T), and neurodegeneration (N)
ATP: adenosine triphosphate
Aβ: amyloid‐β
AβOs: Aβ oligomers
BACE1: β‐secretase 1
BBB: blood–brain barrier
BDNF: brain‐derived neurotrophic factor
CNS: central nervous system
CSF: cerebrospinal fluid
DNA‐: deoxyribonucleic acid
EVs: extracellular vesicles
FDA: US Food and Drug Administration
FNDC5: fibronectin type III domain‐containing protein 5
FTD: frontotemporal dementia
HIV: Human Immunodeficiency Virus
IFN‐γ: interferon‐gamma
IL‐12‐: interleukin‐12
L1CAM: L1 cell adhesion molecule
LPS: lipopolysaccharide
MAPT: microtubule‐associated protein tau
MCI: mild cognitive impairment
MDEVs: microglia‐derived EVs
miRNA: microRNA
MISEV: Minimal information for studies of extracellular vesicles
MMSE: Mini‐Mental State Examination
MRI: magnetic resonance imaging
MSCs: Mesenchymal Stem Cells
NDEVs: neuron‐derived EVs
NIA‐AA: National Institute on Aging–Alzheimer's Association
NVU: neurovascular unit
OligDEVs: oligodendrocyte‐derived
Par‐4: prostate apoptosis response 4
PE: physical exercise
PET: positron emission tomography
p‐tau: tau protein phosphorylated
RNAs: ribonucleic acids
RVG: rabies virus glycoprotein peptide
siRNAs: small interfering RNAs
SVs: synaptic vesicles
TAR: trans‐activation response
TGF‐β: transforming growth factor‐beta
TNF‐α: tumor necrosis factor‐alpha
VaD: vascular dementia
WHO: World Health Organization

## 1 Introduction

Alzheimer's disease (AD) is a neurodegenerative disorder characterized by memory impairment and cognitive decline (2024 Alzheimer's disease facts and figures 2024). According to the World Health Organization (WHO), AD is the leading cause of dementia, accounting for approximately 70% of all dementia cases. It is estimated that around 57 million people worldwide live with dementia, and approximately 33 to 39 million people are affected by AD (Nichols et al. 2019; WHO 2025). Currently, one in nine individuals (10.9%) aged 65 or older is diagnosed with the disease (Rajan et al. 2021).

The main pathological hallmarks of AD include the two neuropathological markers: amyloid plaques, which consist of extracellular deposition of misfolded and aggregated amyloid‐β (Aβ) peptides, and neurofibrillary tangles, which are intraneuronal aggregates composed of the hyperphosphorylation of tau protein (p‐tau) (Knopman et al. 2021). The accumulation of these markers is associated with the progression of the symptoms and the disease severity, as they can be established in brain regions that are critical for cognitive function (Henstridge et al. 2019).

Accumulation of Aβ protein and tau tangles is thought to be fundamental in triggering neuroinflammation and gliosis processes, both of which are also recognized as pathological features of AD (Heneka, van der Flier, et al. 2024). Neuroinflammation is a complex biological process involving the activation of immune cells in response to injury or pathogenic stimuli (Kiraly et al. 2023; Heneka, van der Flier, et al. 2024). Conversely, gliosis is triggered by stimuli and requires the activation and proliferation of non‐neuronal cells, such as astrocytes, microglia, and oligodendrocytes (Kiraly et al. 2023; Heneka, van der Flier, et al. 2024).

Together, neuroinflammation mediators and glial cells respond by altering gene expression, morphology, and the secretion of factors such as cytokines in an attempt to protect neurons against the harmful effects of Aβ peptide and tau tangle deposition (Walters et al. 2016; Heneka, van der Flier, et al. 2024). However, the continued presence of these pathological materials leads to a vicious cycle of brain damage contributing to neurodegeneration, cognitive impairment, and memory loss in AD (Zhang et al. 2024). Given the complexity of AD pathophysiology, a deep understanding of the pathological mechanisms, the identification of biomarkers, and therapeutic approaches is essential to enable healthy aging and improve clinical outcomes. In this context, extracellular vesicles (EVs) have emerged as promising players in neurodegenerative disorders, including AD.

According to the Minimal Information for Studies of Extracellular Vesicles 2023 (MISEV 2023), EVs are micro‐ and nanoparticles delimited by a lipid bilayer membrane, incapable of replicating independently as they do not have a functional nucleus, and can safely carry a variety of biomolecules, including proteins, lipids, small ribonucleic acids (RNAs), and deoxyribonucleic acid (DNA), from the donor cell to the recipient cells. EVs comprise a heterogeneous population, ranging in size from 50 nm to 10 μm (Welsh et al. 2024). They can be found in several body fluids, such as urine, blood, cerebrospinal fluid (CSF), semen, saliva, maternal milk, and amniotic fluid (Ronquist and Brody 1985; Pisitkun et al. 2004; Caby et al. 2005; Admyre et al. 2007; Asea et al. 2008; Vella et al. 2008; Aalberts et al. 2012; Ogawa et al. 2016). Due to the wide variety of particles, MISEV 2023 proposed a classification system based on both size and biological characteristics, given the challenges in distinguishing their subtypes with high specificity. Based on size, EVs are categorized into small vesicles, such as microvesicles and exosomes, and large vesicles, including apoptotic bodies and large oncosomes (Welsh et al. 2024).

Additionally, EVs can also be classified according to their biogenesis: exosomes, which arise from an endosomal origin, and ectosomes, which bud directly from the plasma membrane (Welsh et al. 2024). Considering the complexity and overlap among EV subtypes, MISEV 2023 recommends the use of the umbrella term “extracellular vesicles,” unless it is important to the study to identify the subtype (Welsh et al. 2024). This recommendation aims to ensure consistency and reduce ambiguity in the scientific literature.

**FIGURE 1:**
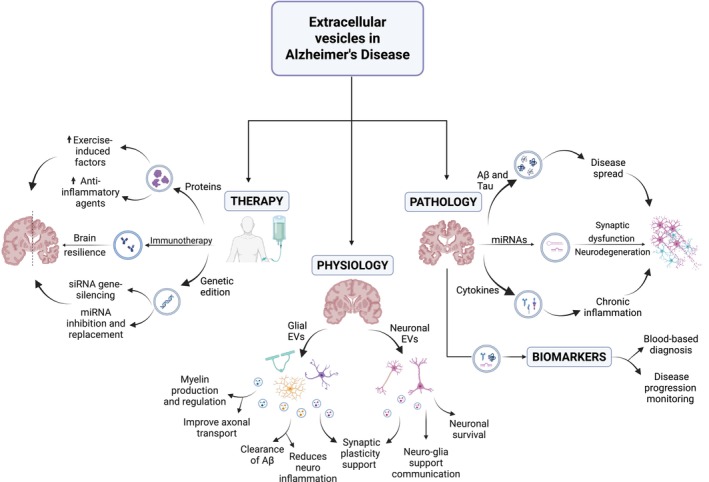
The multifaceted role of extracellular vesicles. Extracellular vesicles (EVs) perform multiple roles in the context of Alzheimer's disease. Under physiological conditions, glial and neuronal EVs contribute to synaptic support, neuroglial communication, attenuation of neuroinflammation, and protein transport. In their pathological role, EVs are implicated in the propagation of amyloid‐beta (Aβ) and tau proteins, synaptic dysfunction, neurodegeneration, and chronic inflammation. As biomarkers, EVs may carry microRNAs (miRNAs), Aβ, tau, cytokines, and other factors, offering valuable tools for the diagnosis and monitoring of disease progression. In the context of EV‐based therapies, approaches such as immunotherapy, anti‐inflammatory agents, gene editing, and the delivery of exercise‐induced factors have been proposed to enhance brain resilience and restore neuronal function. The figure was created with BioRender.com.

Herein, we aim to explore the multifaceted role of EVs in AD, focusing on their contribution to brain physiology and pathology, the mechanisms by which they transport pathological molecules, their spread, their potential in identifying biomarkers, and advancing therapeutic strategies for AD (Figure 1). By shedding light on the diverse roles of EVs, we intend to provide a comprehensive overview of their relevance in the context of AD.

## 2 Extracellular Vesicles as Global Communicators

Cellular communication is a fundamental and highly conserved biological process essential for coordinating functions, responding to environmental challenges, and maintaining homeostasis. Across evolutionary stages, organisms have developed multiple strategies to facilitate intercellular signaling, ranging from simpler contact‐based mechanisms to more complex long‐distance networks, such as the endocrine system. This preserved communication process occurs in several organisms, including bacteria, plants, and humans (Su et al. 2024). Complex organisms rely heavily on intercellular communication, secreting molecules such as proteins, lipids, and nucleic acids. Among the various cell‐to‐cell communication strategies, EVs have emerged as key players, acting as dynamic carriers of intercellular signals (Harding and Stahl 1983; Deatherage and Cookson 2012; Schorey et al. 2015; Jeppesen et al. 2023).

Within the central nervous system (CNS), EVs can mediate neuroprotective actions by being released from CNS cells: neurons, microglia, astrocytes, and oligodendrocytes. These EVs contain important biomolecules that contribute to modulation of gene expression, activation of intracellular signaling pathways, and changes in cellular metabolism. Furthermore, they participate in myelin formation, neurite outgrowth, and neuronal survival. They also can reprogram the cellular phenotype, directly affecting the functional state of the recipient cell (Lachenal et al. 2011; Wang et al. 2014; Antonucci et al. 2012; Williams et al. 2013; Lopez‐Verrilli et al. 2013; Pusic and Kraig 2014; Rajendran et al. 2014; Kanninen et al. 2016). On the other hand, in AD, EVs have been shown to carry and disseminate misfolded proteins such as Aβ and tau, exacerbating neurodegeneration throughout the body (Chivet et al. 2012; Kalani et al. 2014; Tsilioni et al. 2014; Kanninen et al. 2016; Paschon et al. 2016; Thompson et al. 2016; Raghav et al. 2022). A common point between neurodegenerative diseases, such as AD, is the aggregation and formation of misfolded neurotoxic proteins. The progression of AD pathology, for example, may involve the actions of EVs, which facilitate the spread of toxic species in the organism (Vella et al. 2008; Guest et al. 2011; Coleman and Hill 2015).

Initially, EVs were considered a form of “cellular waste disposal,” primarily as a means of eliminating unwanted cell components without significant functional impact on neighboring cells (Ostenfeld et al. 2014). However, in 1983, pivotal studies by Harding and Stahl (1983) and Pan and Johnstone (1983) challenged this notion by introducing the concept of EVs as mediators of physiological processes, demonstrating that EVs were involved in iron transfer in reticulocyte cultures. Although the communicative function of EVs was ignored for years, EVs are currently well‐accepted as messenger carriers within the scientific community.

Given the heterogeneity of biomolecular content and their ability to cross the blood–brain barrier (BBB), EVs are also being recognized in AD clinical diagnosis, as they may indicate the state of the tissue of origin and can be detected in peripheral fluid, including blood (Bongiovanni et al. 2021). Interestingly, EVs are being reported as promising drug carriers due to several advantageous properties, including being inert, non‐immunogenic, biodegradable, and biocompatible. They can be engineered to transport therapeutic molecules such as RNAs, offering novel strategies for AD treatment (El Andaloussi et al. 2013; Rädler et al. 2023).

Taken together, these findings highlight the relevance of EVs as pivotal mediators in different contexts. Hence, this review will focus on the multifaceted roles of EVs in the context of AD, emphasizing their involvement in disease propagation, their potential as diagnostic biomarkers, and their emerging value as targets in therapeutic strategies.

## 3 EVs in Alzheimer's Disease

In pathological contexts, EVs may contribute to disease progression by transporting critical disease‐related factors, thereby facilitating their dissemination (Table 1). In AD, the distribution of Aβ and tau aggregates follows distinct and stereotypical patterns as the disease advances. Aβ deposition initially occurs in the orbitofrontal neocortex and the basal temporal cortex, whereas tau pathology first appears in the locus coeruleus and the transentorhinal cortex before spreading to other regions such as the hippocampus (Braak and Braak 1991; Peng et al. 2020). Interestingly, the tau spreads throughout the brain in a predictable, characteristic pattern (Braak et al. 2006).

**TABLE 1:** Extracellular vesicle‐associated factors in Alzheimer's disease: Pathogenic insights and therapeutic prospects.

Factor	Function	Model	Effects	References
Aβ	Peptide that forms from amyloid precursor protein (APP)	HeLa cells expressing the Swedish mutant of APP (swAPP) using an antibody that specifically supports the β‐cleaved ectodomain of swAPP (sAPPβ)	↑ EVs proteins in the plaques of AD patient brains EVs released from N2a cells contain Aβ peptides	Rajendran et al. (2006)
		Brain sections from the temporal neocortex from *post mortem* AD brains	↑ levels of AβOs in EVs from AD patients' brains ↓ EVs = ↓ AβOs and the related toxicity	Sardar Sinha et al. (2018)
		Release in co‐cultures of cortical astrocytes, neurons, and oligodendrocytes	↑ levels of Aβ = ↑ p‐tau, prostate apoptosis response 4, and ceramide	Söllvander et al. (2016)
Tau	Microtubule‐associated protein essential for the stability and organization of the neuronal cytoskeleton	Human brain‐derived extracellular vesicles isolated from patients with Alzheimer's disease, prodromal Alzheimer's disease, and control cases without dementia	↑ epitope‐specific tau oligomers in ADEVs ↑ EVs transferring and misfolding tau in vitro	Ruan et al. (2021)
		Brain EVs from individuals with AD	EVs from the brains of individuals with AD are associated with truncated tau	Fowler et al. (2025)
		Isolated and characterized murine BD‐EVs from a transgenic mouse model of tauopathy	Presence of tau in BD‐EVs may induce lesions in vivo	Leroux et al. (2022)
APP	Transmembrane protein that serves as the precursor to Aβ	EVs from N2a cells expressing human APP with the autosomal dominant Swedish mutation	↑ CTF‐α in EVs secreted by cortical neurons; APP‐containing EVs bind to neurons, and after endocytosis, the CTFs of the EVs are processed by γ‐ secretase in the recipient cell	Laulagnier et al. (2018)
APP‐CTFs	CTFs result from the cleavage of APP by different enzymes	EVs were purified from cell media or brains of mice expressing C99 or APPswedish treated with vehicle or D6	EVs are enriched in APP‐CTFs and contain high molecular weight APP‐CTFs, the levels of which are increased by γ‐secretase inhibition	Lauritzen et al. (2019)
		EVs isolated from freshly removed mouse brains and from frozen mouse and human brain tissues	Secreted brain EVs contain flAPP, APP‐CTFs, and Aβ	Perez‐Gonzalez et al. (2012)
BDNF	Neurotrophin associated with the development, differentiation, and survival of neurons	Ischemic stroke mouse model treated with mesenchymal stem cell‐derived small extracellular vesicles overexpressing BDNF	↑ neurogenesis, angiogenesis, and synaptic plasticity ↓ neuroinflammation ↑ functional behavior	Zhou et al. (2023)
		Major depression mouse model treated with RVG‐modified exosomes overexpressing BDNF	↑ neurogenesis and synaptic plasticity ↓ neuroinflammation ↓ depression‐like behaviors	Liu, Shen, et al. (2024)
		Parkinson's disease mouse model treated with human umbilical cord mesenchymal stem cell‐ derived exosomes overexpressing BDNF	↑ neuronal survival ↓ p‐Tau hyperphosphorylation ↓ Parkinson's disease‐like behaviors	Wang et al. (2024)
Curcumin	Plant‐based chemical linked to antioxidant and anti‐inflammatory properties	LPS‐induced brain inflammatory and autoimmune encephalitis mouse model treated with curcumin‐encapsulated exosomes	↑ leukocyte apoptosis in the brain of LPS‐induced mice ↓ IL‐1β protein and mRNA expression in the brain of LPS induced and autoimmune encephalitis mice	Zhuang et al. (2011)
miR‐133b	miRNA related to the development of skeletal muscle and to neuronal protection	Intracerebral hemorrhage rat model treated with mesenchymal stem cell‐derived exosomes overexpressing miR‐133b	↑ ERK1/2 and CREB phosphorylation in brain tissues ↓ neuronal apoptosis and neurodegeneration	Shen et al. (2018)
miR‐25‐3p	miRNA involved in the regulation of cell growth, migration, and apoptosis	Cerebral ischemia mouse model treated with adipose‐derived mesenchymal stem cell‐derived extracellular vesicles with an anti‐miR‐25‐3p oligonucleotide	↓ neuronal density in ischemic lesion site compared to EVs without anti‐miR‐25‐3p ↑ autophagic flux in ischemic striatum compared to EVs without anti‐miR‐25‐3p ↓ motor coordination compared to EVs without anti‐miR‐25‐3p	Kuang et al. (2020)
BACE1 siRNA	The BACE1 gene encodes the BACE1 protein, enzyme that cleaves the amyloid precursor protein to produce amyloid‐β	Wild‐type mice treated with dendritic‐cell‐derived bone marrow‐derived exosomes fused with RVG and electroporated with BACE1 siRNA	↓ BACE1 protein in cortex ↓ amyloid‐β 42 levels in cortex no significant changes in cytokines in serum	Alvarez‐Erviti et al. (2011)
BACE1 and caspase‐3 siRNA	The caspase‐3 gene encodes the caspase 3 protein, a protease that participates in the apoptosis process	Alzheimer's disease mouse model treated with lesion‐recognizing nanoparticles containing RVG peptide‐fusioned mesenchymal stem cell‐derived exosomes loaded with BACE1 and caspase‐3 siRNA	↓ BACE1 and caspase‐3 levels ↓ amyloid‐β plaques and reactive astrocytes ↑ cognitive function and neuronal integrity	Li et al. (2023)

EVs are increasingly recognized as key mediators of intercellular communication, particularly through their role in transporting disease‐related factors associated with AD (Vingtdeux et al. 2012). Although the exact mechanisms of propagation of these disease‐related factors are not fully understood, EVs have been proposed as one of the contributors to AD propagation (Hill 2019). Interestingly, accumulating evidence has demonstrated altered secretion and function of EVs during the progression of AD (Gallart‐Palau et al. 2020), and that inhibiting EV release significantly alleviates the AD phenotype (Asai et al. 2015), highlighting the crucial role of EVs in the pathogenesis of AD (Su et al. 2021).

### 3.1 Pathological Roles of EVs in AD

Previous studies have shown that tau is primarily secreted in its free form, with a minor fraction found in EVs, as observed in the CSF and blood of both control subjects and AD patients (Arai et al. 1998; Saman et al. 2012; Zetterberg et al. 2013; Fiandaca et al. 2015; Ruan and Ikezu 2020; Brunello et al. 2020). Furthermore, hyperphosphorylated assembled tau is associated with EVs isolated from the brain tissue of individuals with AD (Ruan et al. 2021; Fowler et al. 2025) (Table 1). In another study, tau filaments are tethered within brain EV in AD (Fowler et al. 2025) (Table 1), suggesting a potential pathogenic role of tau in the development of the disease (Figure 1). It has also been observed in humans that EVs enriched from biological fluids, specifically brain‐derived EVs, can facilitate the prion‐like propagation of tau pathology across various tauopathies (Leroux et al. 2022) (Table 1).

In contrast to tau pathology, amyloid plaques display a less predictable spreading pattern (Braak and Braak 1991). The research focus has shifted toward soluble Aβ oligomers (AβOs) as being the main neurotoxic Aβ conformation capable of inducing synaptic dysfunction (Mroczko et al. 2018). These oligomers also appear to be related to the proposed hypothesis of prion‐like propagation of Aβ pathology (McAllister et al. 2020). In 2006, Rajendran demonstrated that Aβ can be secreted from cells in association with exosomes, and EVs accumulate in plaques of AD patients Figure 1. This suggests that exosomes may contribute to the pathogenesis of AD by facilitating the intercellular transport of Aβ (Rajendran et al. 2006) (Table 1).

Another study demonstrated that neuronal exosomes may act as vehicles for the intercellular transport of amyloid‐beta precursor protein (APP) and its catabolites (Laulagnier et al. 2018). Furthermore, in vivo experiments revealed the presence of oligomeric amyloid precursor protein‐derived C‐terminal fragments (APP‐CTFs) in AD mouse models, the levels of which are selectively enriched in endolysosomal compartments, including EVs, and amplified by γ‐secretase inhibition (Lauritzen et al. 2019) (Table 1).

A previous finding showed that EVs isolated from the brains of AD‐transgenic mice contained APP, APP‐CTFs, and Aβ. The levels of APP and APP‐CTFs within the EVs were higher compared to those in EVs isolated from non‐transgenic mice. APP‐CTFs were enriched in EVs compared to cell lysates, but no difference in the total amount of EVs was reported (Perez‐Gonzalez et al. 2012). Importantly, a prior study of our group demonstrated that EVs extracted from the brains of postmortem patients with AD and frontotemporal dementia (FTD) negatively affected memory performance in mice. These findings suggest that, in addition to their role in molecular transport, EVs may contribute to the spread of pathological proteins, thus promoting memory impairment in AD and FTD. Furthermore, we reported disease‐specific differences in the protein cargo of brain‐derived EVs in AD and FTD (Bodart‐Santos et al. 2023).

Neurons are electrically excitable cells that communicate with other cells through neurotransmission and function as the primary functional units of the CNS (Xia et al. 2022). Neuron‐derived EVs (NDEVs) have garnered considerable attention in AD research, as they serve as carriers of pathological molecules. NDEVs isolated from the blood of AD patients have been shown to contain significantly higher levels of Aβ1–42 and p‐Tau, as well as alterations in lysosomal proteins, compared to those from healthy individuals (Goetzl et al. 2015; Abner et al. 2016) (Figure 1).

An immunoadsorption‐based strategy using an immunocapture analysis targeting proteins highly expressed in neurons, such as L1 cell adhesion molecule (L1CAM) antibody, has been developed to purify NDEVs from blood samples. This approach has raised concerns about the utility of L1CAM as a marker for NDEVs, as L1CAM is not exclusively expressed in neurons (Norman et al. 2021; Gomes et al. 2022). Therefore, advanced methodologies are needed to purify NDEVs from blood samples while minimizing contamination. Furthermore, studies have suggested that NDEVs act as vehicles facilitating the pathological spread of AD‐related molecules between brain cells and may promote amyloid fibril formation by Aβ within the CNS (Sardar Sinha et al. 2018) (Table 1). Interestingly, other studies have found levels of Aβ peptides and tau species in L1CAM+ EVs. Additionally, ATP1A3+ EVs have been shown to carry Aβ peptides and t‐tau, as well as p‐tau181. ATP1A3 represents a neuronal‐specific subunit of the Na^+^/K^+^‐ATPase (Fiandaca et al. 2015; You et al. 2023; Rocha et al. 2024). These findings collectively suggest that EVs not only reflect disease pathology but may actively participate in its propagation. A recent review by our research group highlighted the potential of EVs as biomarkers while also addressing key challenges, including their relatively low abundance, the absence of standardized and validated isolation protocols, and the difficulty in identifying specific targets for NDEV immunocapture (Rocha et al. 2024).

Neuronal damage induced by the content of NDEVs is likely mediated by the transfer of AD‐associated pathogenic molecules, such as amyloid precursor protein (APP) and toxic AβOs (Sardar Sinha et al. 2018) (Table 1). Compared to NDEVs isolated from the plasma of healthy individuals, those derived from the blood of AD patients exhibit significant neurotoxic effects on cultured rat cortical neurons, as evidenced by a reduction in cell viability (Nogueras‐Ortiz et al. 2020). Additionally, another study has shown that excessive Aβ can increase the expression of p‐tau, prostate apoptosis response 4 (Par‐4), and ceramide, thereby promoting the formation of enlarged endosomes and the subsequent release of astrocyte‐derived EVs (ADEVs) in a co‐culture system (Söllvander et al. 2016) (Table 1).

In AD, it has been found that microglia‐derived EVs (MDEVs) directly transport classic AD pathogenic factors, including Aβ and tau, between cells. Extracellular Aβ42 protofibrils can be internalized by microglia and subsequently transported into MDEVs (El Andaloussi et al. 2013; Gouwens et al. 2018). Therefore, EVs display both pathological and potentially beneficial effects, indicating dynamic changes in EVs throughout the progression of AD.

### 3.2 Neuroinflammation Roles of EVs in AD

Neuroinflammation, characterized by immune responses within the CNS, stands as a central player in various neurological disorders, such as AD, multiple sclerosis, Parkinson's disease, and hepatic encephalopathy (Heneka et al. 2014; Cabrera‐Pastor et al. 2019). Therefore, elucidating the mechanisms that drive neuroinflammation is crucial for the development of effective therapeutic strategies against these debilitating conditions (Gao and Hong 2008). The EVs regulate each other and influence the function of CNS‐resident cells. Consequently, understanding the functional role and underlying mechanisms of EVs in the onset and resolution of these neuroinflammatory diseases is gaining increasing attention.

EVs containing toxic proteins are internalized by both local and distant neurons, contributing to neuronal loss and suggesting a mechanism that underlies the spread of toxic proteins in the neurodegenerative brain. Along with neurons, the propagation of toxic proteins can also be mediated by microglia, whose activation is a hallmark of neurodegenerative diseases. Microglia have been shown to contribute to the spread of Aβ and tau through exosome secretion (Joshi et al. 2014; Asai et al. 2015). Blocking exosome synthesis in vivo reduced amyloid plaque formation and tau propagation, respectively, in the corresponding mouse models (Dinkins et al. 2014; Ruan et al. 2020).

In an in vitro AD model, it was shown that EVs can be internalized by microglia, leading to both acute and delayed upregulation of tumor necrosis factor‐alpha (TNF‐α) and other pro‐inflammatory factors that drive neuroinflammation through the delivery of miRNAs to the microglia (Fernandes et al. 2018) (Figure 1). Glial cells, such as astrocytes, oligodendrocytes, and microglia, not only orchestrate inflammatory responses to infections or diseases but also continuously provide neurotrophic support and play a role in synaptic remodeling and pruning. In addition to traditional direct cell‐to‐cell interactions and the paracrine effects of secreted molecules, glial cells and neurons communicate through the release and uptake of EVs. This form of communication facilitates coordinated regulation across long distances (Budnik et al. 2016; Krämer‐Albers 2022). Notably, microglia depend significantly on mobile vesicles to propagate cytokine‐mediated inflammatory signals across distant areas of the brain (Frühbeis, Fröhlich, Kuo, Amphornrat, et al. 2013).

Understanding the complex roles of EVs in neuroinflammation is essential for unraveling the pathophysiology of neurological disorders and exploring potential therapeutic strategies targeting EV‐mediated processes. It is important to recognize EVs as a signaling system that crosses boundaries, as they provide valuable insights and hold potential for more effective biological therapies. However, further research is required to fully comprehend the mechanisms.

## 4 EVs: Key Players in CNS Health

In the brain, despite the essential role of neurons in transmitting nerve impulses and responding to stimuli through membrane potential changes, there are a variety of cell types that interact and support each other, especially glial cells (Verkhratsky et al. 2011). These non‐neuronal cells are essential for brain homeostasis and include astrocytes, which form the BBB and therefore regulate the passage of molecules between the blood and the CNS; microglia, which participate in the brain's immune defense through phagocytosis; and oligodendrocytes, responsible for producing the myelin sheath in the CNS (Perea et al. 2014; Verkhratsky et al. 2025). The interaction between neurons and glial cells is fundamental for the metabolic and functional regulation of the nervous system, including synaptic efficiency (Araque et al. 1999; Iadecola and Nedergaard 2007; Perea et al. 2009).

Intracerebral communication has been described as involving the participation of EVs, which are released by all cell types in the brain. The content of these vesicles varies according to the donor cell and may contain specific proteins (Fauré et al. 2006; van Niel et al. 2006; Turola et al. 2012; Rufino‐Ramos et al. 2023). Neurons perform their classical function of neurotransmitter transmission, which enables the propagation of neuronal activation at chemical synapses. This process involves the release of vesicles, known as synaptic vesicles (SVs), from presynaptic neurons to postsynaptic neurons. These vesicles are slightly smaller, 35 to 55 nm, and have only 1 lipid membrane (Cooney et al. 2002). SVs primarily contain neurotransmitters and are regulated by the neurons themselves; they can either be recycled or released via exocytosis, depending on the signaling pathways activated within the cells (Kwon and Chapman 2011). In addition to synaptic vesicles, neurons are also capable of releasing other types of vesicles, such as exosomes, which carry a diverse range of molecular cargo, including RNAs and miRNAs (Figure 1). This suggests a broader capacity for modulating biological processes through vesicle‐mediated communication (Schnatz et al. 2021; Chivet et al. 2012).

### 4.1 Neuronal EVs

The hypothesis that NDEVs regulate synaptic activity is gaining support, and evidence demonstrates that there is a selective absorption of vesicles from the hippocampus of mice by other neurons in vitro (Chivet et al. 2014). Different studies found that neurons can release EVs that promote synaptic pruning by microglia and that the process is regulated by calcium influx and glutamatergic synaptic activity (Lachenal et al. 2011; Bahrini et al. 2015). According to Antoniou and collaborators, the brain‐derived neurotrophic factor (BDNF) can stimulate the release of NDEVs that modulate neural network connectivity (Antoniou et al. 2023) (Figure 1). In line with the regulation of synaptic plasticity, vesicles derived from human neuroblasts are resistant to RNAse degradation and modulate miRNA concentrations in neurons (Goldie et al. 2014). In a separate study, NDEVs were found to carry miR‐124a, which is taken up by astrocytes, leading to increased expression of glutamate transporters and, consequently, modulation of synaptic activity (Morel et al. 2013).

Neurons are also capable of releasing vesicles that are taken up by glial cells, where they stimulate the regeneration of nervous tissue and promote axonal growth (Goncalves et al. 2015). NDEVs collected from rat neuronal culture media have been shown to enhance microglial viability and promote an anti‐inflammatory phenotype, thereby improving microglial performance in inflammatory responses (Peng et al. 2021).

Given their diverse physiological roles, NDEVs may contribute positively to mechanisms that are disrupted in AD. The ability of NDEVs to regulate synaptic plasticity, promote glutamate homeostasis, and influence microglial phenotype suggests that these vesicles may help preserve synaptic integrity and reduce neuroinflammatory damage in the early stages of the disease. Moreover, the transport of regulatory molecules such as miRNAs and neurotrophic factors via NDEVs may modulate the glial response, offering a potential endogenous mechanism to counteract neurodegeneration in AD.

### 4.2 Microglia‐Derived EVs

Studies have shown that vesicles derived from the brains of mice are more efficiently phagocytosed by primary microglial cultures than by astrocytes and are directed to endolysosomes for processing. Furthermore, these vesicles, when associated with α‐synuclein oligomers, misfolded proteins related to Parkinson's disease, are degraded in lysosomes, whereas free oligomers accumulate within cells (Pantazopoulou et al. 2023). These results suggest that microglia not only internalize and guide EV processing but also play a role in the clearance of misfolded proteins. In AD, microglia are activated by AβOs and initiate an important process of clearance and removal of these molecules, playing an essential role in protecting against disease‐related damage (Lee and Landreth 2010; Heckmann et al. 2019; Cai et al. 2022). However, as the disease progresses, microglia remain chronically activated, which may lead to dysfunction and excessive release of cytokines, potentially further impairing tissue function. Notably, microglia frequently utilize the EV system for the excretion by apoptotic bodies as well as the release of molecules such as cytokines via exosomes and microvesicles. Some studies suggest that statins may help reduce Aβ peptide production, and more recently, it has been demonstrated that this process may occur through the stimulation of microglial degradation of these peptides via the release of insulin‐degrading enzyme through EVs (Tamboli et al. 2010).

Another fundamental role of microglia is supporting neuronal synapses. In vitro studies have demonstrated that MDEVs modulate synaptic activity, an effect dependent on sphingolipid metabolism, which is implicated in enhancing excitatory neurotransmission (Antonucci et al. 2012). Additionally, MDEVs contain miRNAs capable of inducing either pro‐inflammatory or anti‐inflammatory profiles in neurons, thereby regulating synaptic protein levels (Prada et al. 2018) (Figure 1). Synaptic support is critically important in AD, as there is a reduction in neurotransmitter release due to impaired neuronal activity. Many of the current pharmacological treatments for the disease aim to enhance synaptic function. Therefore, investigating the role of MDEVs in this context may prove highly valuable.

It has also been found that MDEVs exposed to mesenchymal stem cells induce oligodendrocyte remyelination (Lombardi et al. 2019). Moreover, adenosine triphosphate (ATP), a primary energy molecule, serves as a key trigger for the release of EVs by microglia while also promoting astrocyte activation (Bianco et al. 2005; Drago et al. 2017). These findings underscore the bidirectional regulatory interplay between microglial EVs and other cell types.

### 4.3 Oligodendrocyte‐Derived EVs

The function of oligodendrocyte‐derived EVs (OligDEVs) has also been the subject of research, and it has been revealed that these EVs contain proteins involved in the removal of redundant myelin (Krämer‐Albers et al. 2007). Another study supporting the idea that OligDEVs regulate myelin production showed that these vesicles carry growth‐inhibitory factors (Bakhti et al. 2011). The OligDEVs are critical for sustaining axonal transport and metabolic homeostasis in neurons. Under stress conditions, OligDEVs enhance axonal transport in hippocampal neurons (Frühbeis et al. 2020). Additionally, oligodendrocytes have been shown to release EVs enriched with ferritin heavy chain, which acts as an antioxidant, preventing ferroptosis‐induced neuronal death in aged mice (Mukherjee et al. 2020). Beyond their role in demyelinating diseases, the neuroprotective properties of OligDEVs may also support CNS function and promote neuronal resilience in neurodegenerative disorders, such as AD. The regulation of myelin production is critically important. Disruption of myelin synthesis in aged animal models of AD has been shown to impair Aβ processing and promote the accumulation of amyloid plaques in their brains (Depp et al. 2023). Reduced myelin levels have also been reported in other AD models and confirmed through postmortem analyses of human brain tissue (Mitew et al. 2010). A study showed that stimulating myelination improves the cognitive deficit of transgenic animals for AD (Chen et al. 2021). These findings suggest that the myelination support provided by OligDEVs may be important for preserving neuronal function and preventing the progression of AD.

These vesicles are selectively internalized by microglia with an anti‐inflammatory profile and can mediate neuroprotective functions. The glutamate release from neurons may also regulate the release of OligDEVs (Fitzner et al. 2011; Frühbeis, Fröhlich, Kuo, and Krämer‐Albers 2013). Therefore, OligDEVs may not only regulate neuronal myelination but also support other neural cells while being regulated by signaling from other cells.

### 4.4 Astrocyte‐Derived EVs

Finally, ADEVs play diverse roles in CNS regulation, including modulating microglial phagocytic function under stress conditions and inducing phagocytosis when carrying specific small interfering RNAs (siRNAs) (Hu et al. 2018) (Figure 1). Additionally, these EVs help regulate the peripheral inflammatory response to neuroinflammation in mice, mediating periphery‐to‐brain communication (Dickens et al. 2017). Human ADEVs also carry neuroprotective factors and are directed to neurons, protecting against oxidative stress (Pascua‐Maestro et al. 2019). Research has also revealed that, in response to ATP or IL‐10, these vesicles contain proteins involved in dendritic branching and the regulation of synaptic transmission (Datta Chaudhuri et al. 2020). The astrocytic role in regulating inflammation and combating oxidative stress is particularly important in the early stages of AD, as the neurotoxins released during disease progression lead to chronic inflammation, resulting in impaired neuronal metabolism and increased oxidative stress. In addition, ADEVs may also support synaptic function. Similar to MDEVs, ADEVs can contribute to the restoration of neuronal excitability, which is compromised in AD. It has been shown that astrocytes release vesicles containing excitatory amino acid transporters ex vivo in spinal cord explants from rats, modulating synaptic activity (Gosselin et al. 2013). Another study demonstrated that ADEVs from rat hippocampal cultures can carry a microRNA (miRNA) responsible for regulating dendritic complexity (Luarte et al. 2015). Patel and Weaver showed that ADEVs enhance spine and synapse formation by primary cortical neurons (Patel and Weaver 2021). These findings show the importance of astrocyte EVs in the overall support of the nervous system and its response to internal and external stimuli.

Collectively, these findings highlight the relevance of neuron‐ and glia‐derived EVs in intercellular communication and CNS homeostasis, as well as their potential influence on neuroprotection and brain resilience in AD.

## 5 EVs as Blood‐Based Biomarkers

The small size and biocompatibility of EVs confer several advantages, including the ability to overcome biological barriers such as the BBB (Jeppesen et al. 2023; Ravichandiran et al. 2024). The BBB is one of the most selective and complex barriers in the body, playing a key role in protecting the brain from potential threats while maintaining brain homeostasis. It is composed of monolayers of endothelial cells that regulate the nutrient supply and waste elimination and is supported by the neurovascular unit (NVU), which includes pericytes, astrocytes, oligodendrocytes, microglia, and neurons. All these elements influence the maintenance of BBB phenotype and integrity (Saint‐Pol et al. 2020; Dotiwala et al. 2023).

Recent studies have investigated the capacity of EVs to cross the BBB and modulate brain functions. Alvarez‐Erviti and colleagues demonstrated that EVs derived from primary dendritic cell cultures successfully crossed the BBB and delivered siRNA to reduce the expression of specific proteins in the mouse cortex, such as β‐secretase 1 (BACE1), an aspartyl protease involved in Aβ peptide generation (Alvarez‐Erviti et al. 2011). Conversely, research using a zebrafish model showed that brain‐derived EVs or EVs carrying miR‐132 modulated the expression of adherent junction‐related proteins, and their inhibition led to an increase in BBB permeability (Xu et al. 2017). Moreover, Haney and colleagues demonstrated that EVs injected intravenously with DIL‐labeled exosomes revealed a wide distribution of exosomes throughout the brain (Haney et al. 2015). Together, these findings suggest the dual nature of EVs, which are not only players capable of influencing the crosstalk between the periphery and the brain but also biomarkers reflecting both the physiology and pathophysiology of the CNS, serving as a potential window to the brain.

Biomarkers play a crucial role in the context of AD, as neuropathological changes begin years before the manifestation of the clinical symptoms. Therefore, biomarker detection offers predictive, diagnostic, and prognostic value, which is essential for developing preventative strategies to slow cognitive decline. Several biomarkers that indicate the presence of amyloid plaques, tau tangle formation, and neuronal damage have been proposed for AD (Gunes et al. 2022; Grande et al. 2025; Santos et al. 2025). The 2018 National Institute on Aging–Alzheimer's Association (NIA‐AA) research framework defines AD biologically, based on three core pathological processes: amyloid deposition (A), tau pathology (T), and neurodegeneration (N) (ATN) (Jack Jr. et al. 2018).

Currently, the diagnosis of AD depends on the measurement of Aβ42, total tau, or p‐tau protein level in CSF, obtained via an invasive lumbar puncture, accompanied by neuroimaging techniques, such as positron emission tomography (PET) and magnetic resonance imaging (MRI) (Jack Jr. et al. 2024). While these methods have high specificity and sensitivity for detecting AD biomarkers, their widespread clinical application is hindered by high costs and invasiveness, which restricts their routine use and capacity for monitoring in longitudinal follow‐up. Consequently, there is growing interest in minimally invasive alternatives, such as blood‐based biomarkers. Recently, and promisingly, the US Food and Drug Administration (FDA) approved the first in vitro diagnostic kit for early AD detection using blood samples, the Lumipulse G p‐Tau217/β‐amyloid 1–42 Plasma Ratio. Quantification of p‐tau 217 and β‐amyloid 1–42 levels using this new Lumipulse test correlates with the presence or absence of amyloid plaques in the brain, offering a less invasive and more accessible diagnostic option (U.S. FDA 2024).

Indeed, blood‐based biomarker assays have become very sensitive for detecting Aβ42/40 and p‐tau. However, this approach is still being refined, as peripheral sources may be impacting the correlation strength of these protein levels in blood and CSF. For example, Aβ42/40 in CSF can be identified with a reduction of up to 50%; while in blood, a decrease of up to 20% of circulating Aβ42 is demonstrated (Olsson et al. 2016). Furthermore, the potential peripheral confounding also has an effect, as it impedes specific brain analysis, thus limiting their ability to identify the specific molecular alterations occurring in AD brain cells and reducing even more the presence of brain‐derived proteins (Cleary et al. 2025). In this context, EVs have been investigated as promising tools for assessing CNS biomarkers. The source of biomarkers, such as proteins, nucleic acids, miRNAs, and lipids, is capable of conveying useful information for the clinic, reflecting the molecular signature of the disease.

EVs secreted by CNS cells can be detected in the bloodstream, providing a non‐invasive “liquid biopsy” of the brain (Cleary et al. 2025). Numerous studies have reported that NDEVs contain AD‐related proteins, including Aβ, total tau, p‐tau217, p‐tau181, and miRNA, making them promising candidates for early diagnosis and disease monitoring (Aharon et al. 2020; Badhwar and Haqqani 2020; Kumar et al. 2023; Cleary et al. 2025). These NDEV profiles in AD patients, when compared to controls, have been correlated with synaptic dysfunction and neuroinflammation, hallmark features observed in AD patients (Singh et al. 2024). Appealingly, NDEVs carrying p‐tau217 from AD patients were significantly decreased compared to controls, with excellent diagnostic discriminatory power (AUC ~ 0.93–0.96) (Eren et al. 2022). Moreover, EVs have been found to carry these proteins in the preclinical stages of AD, further underscoring their potential utility in identifying individuals at risk or in the early stages of the disease (Berriel Pinho et al. 2024; Taha 2025).

In addition to carrying disease‐related factors, miRNAs are also potential biomarker candidates within EVs due to their stability in extracellular circulation and their involvement in modulating different biological processes in both pathological and non‐pathological states, including neuronal proliferation, development, and synaptic plasticity in the brain (Zhang et al. 2015; Taha 2025). In the AD brain, Satoh et al. reported 13 upregulated miRNAs and 14 downregulated miRNAs (Satoh et al. 2015). Among these, miRNA‐193b has caught attention, as it has been detected in the hippocampus of AD model mice. A previous study reported that miRNA‐193b downregulates APP, but total EV levels in miRNA‐193b are found to be increased (Liu et al. 2014; Yang et al. 2018). This paradox suggests that a decrease of miRNA‐193b in neurons might be associated with its increased secretion via exosomes.

Indeed, Cheng and colleagues showed that exosomes in biofluids promote a protective environment and enriched population of miRNAs, offering a feasible approach for determining biomarkers for AD (Cheng et al. 2015). Moreover, a machine learning model identified 7 AD‐associated miRNAs capable of predicting AD status (Lugli et al. 2015). Recently, Reho et al. identified 14 miRNAs associated with AD risk in a multiethnic cohort, with many of them targeting the microtubule‐associated protein tau (MAPT) gene (Reho et al. 2025). Altogether, these findings highlight the potential of EV‐associated miRNAs to serve as diagnostic tools.

Interestingly, Li et al. explored whether combining protein miRNA analysis within EVs could also have potential in discriminating AD from mild cognitive decline (MCI) and vascular dementia (VaD). Notably, they found that hsa‐miR‐1306‐5p, hsa‐miR‐93‐5p, hsa‐miR‐424‐5p, hsa‐3065‐5p, and P‐S396‐tau were differentially expressed across diagnostic groups, suggesting a potential integrative EV‐based biomarker signature for distinguishing sporadic AD from MCI and VaD (Li, Chen, et al. 2020). In summary, the presence of both disease‐associated proteins and regulatory miRNAs within NDEVs supports their potential in the diagnostic field, as it enables the identification of minimally invasive brain‐specific biomarkers for early detection, safer disease monitoring, and discriminating between dementias.

On the other hand, despite these advantages, several challenges remain. One major limitation is the lack of standardized methods for EV isolation and characterization. Although L1CAM is highly expressed in the brain, its presence in peripheral tissues like the kidney and reproductive organs raises concerns about specificity. Alternative markers such as SNAP25 and ATP1A3 are being investigated to improve the selective capture of NDEVs (Rocha et al. 2024). Importantly, EV‐based biomarkers align with the ATN classification framework and represent a transformative approach to diagnosing and managing AD. By enabling earlier and more accurate detection of neuropathological changes, EVs have the potential to shift clinical paradigms from late‐stage symptom management to proactive intervention strategies.

## 6 EVs as Neuroprotective Mechanism: Findings and Perspectives

As the multifaceted roles of EVs remain to be elucidated, questions have emerged regarding their potential applications in enhancing health, particularly in the context of diseases. EVs have been identified as a promising vehicle for transporting molecules of interest to the brain to silence or overexpress specific factors that may alleviate symptoms, promote brain resilience, and improve overall health.

### 6.1 Proteins

One potential approach to mitigate AD symptoms involves modulating the expression of neuroprotective proteins within EVs. Given that a healthy lifestyle has been linked to longevity and healthy aging, physical exercise (PE) has been extensively studied as a prospective therapeutic strategy for AD (2024 Alzheimer's disease facts and figures 2024; Tari et al. 2025). The beneficial effects of PE include functional and morphological adaptations in both peripheral and central biological systems. In the CNS, PE has been shown to enhance cognitive function by promoting synaptic plasticity, increasing cerebral vasculature, reducing neuroinflammation, preventing age‐related brain volume reduction, and increasing hippocampal volume (Colcombe et al. 2006; Ding et al. 2006; Erickson et al. 2011; García‐Mesa et al. 2011; Braskie et al. 2014; Ten Brinke et al. 2015; Maass et al. 2016; Ribeiro et al. 2025). Collectively, these mechanisms contribute to a reduced risk of developing neurological disorders, underlining the importance of PE in AD treatment.

The beneficial effects of PE are mediated through exerkines, molecules released into the bloodstream that can cross the BBB and promote neuroprotection (Trovato et al. 2019; Chow et al. 2022). Although exerkines can be secreted in their free form, evidence suggests that their effects are modulated via EV secretion. Accordingly, PE has been associated with increased EV circulation. In a study conducted by Frühbeis et al., EV concentration in human plasma was found to be uplifted immediately after aerobic exercise, subsequently declining 90 min post‐exercise (Frühbeis et al. 2015). The role of EV‐associated exerkines in therapeutic applications was initially proposed by Safdar et al., presented for metabolic diseases. The research demonstrated that ALG‐2 interacting protein X (ALIX), an exosome marker, was depleted in skeletal muscle cells after one hour of endurance exercise, suggesting that skeletal muscle secretes EVs in response to PE stimuli (Safdar et al. 2016). Similarly, Whitham et al. observed an increase in EVs in human plasma after one hour of aerobic exercise, and 35 new exerkines were described (Whitham et al. 2018). Together, these findings support the hypothesis that EVs play a critical role in mediating the physiological benefits of PE.

Exercise‐derived EVs have been studied for their protective functions in the CNS. In a preclinical model of cerebral ischemia, it was demonstrated that exercise prior to stroke induced neuroprotection and was mediated by skeletal muscle‐derived EVs (Huang et al. 2024). Additionally, intranasal administration of exercise‐derived EVs reduced resting energy expenditure in a transgenic mouse model of AD, although no cognitive improvement was observed (Fuller et al. 2025). Considerable interest has been directed toward identifying the specific exerkines responsible for the beneficial effects of PE, with numerous studies reporting various proteins and metabolites implicated in neuroprotection (Safdar et al. 2016; Whitham et al. 2018; Chong et al. 2024). Among these, BDNF, fibronectin type III domain‐containing protein 5 (FNDC5)/irisin, cathepsin B, and lactate have been identified as key players in neuroprotective mechanisms (Moon et al. 2016; El Hayek et al. 2019; Lourenco et al. 2019; Valenzuela et al. 2020; Rody et al. 2022). These findings illuminate the molecular pathways underlying the EV‐mediated effects of PE on CNS function. Consistent with this, overexpression of BDNF in EVs has been shown to enhance neurogenesis and synaptic plasticity and improve behavior in mouse models of ischemic stroke, depression, and Parkinson's disease (Zhou et al. 2023; Liu, Chen, et al. 2024; Wang et al. 2024) (Table 1).

Despite the complexity of physical exercise and its multifaceted impact on metabolic pathways, elucidating its underlying mechanisms may offer valuable insights for developing novel therapeutic strategies to mitigate cognitive impairment associated with AD (Figure 1) and related conditions, such as hypertension, obesity, diabetes, depression, and cardiovascular diseases (Daviglus et al. 2011; Selkoe 2012; Ledo et al. 2013; De Felice et al. 2014, 2024 Alzheimer's disease facts and figures 2024).

In addition to exercise‐associated proteins, anti‐inflammatory agents represent an innovative strategy for the development of effective treatments for AD. Curcumin, a plant‐derived compound known for its anti‐inflammatory properties, was encapsulated in exosomes and used to treat neuroinflammatory diseases, including lipopolysaccharide (LPS)‐induced brain inflammation and autoimmune encephalitis. Intranasal administration of these exosomes demonstrated protective effects against such diseases. The exosomes were preferentially internalized by microglia, where they induced apoptosis, thereby reducing the population of activated inflammatory microglial cells (Zhuang et al. 2011) (Table 1).

The encapsulation of anti‐inflammatory agents within EVs represents a promising strategy to optimize their biological stability and facilitate their transport across the BBB, thereby contributing to the reduction of the neuroinflammatory state associated with AD (Figure 1).

### 6.2 miRNAs and siRNAs

EV‐based therapeutic strategies involving nucleic acids, such as miRNAs and siRNAs, have also attracted significant interest. miRNAs are known to regulate multiple biological processes (Bartel 2004); whereas siRNAs, though less prevalent in mammals, are widely utilized in biomedical research (Dana et al. 2017). Both miRNAs and siRNAs are small non‐coding RNAs that function as gene regulators, typically through mRNA inhibition (Carthew and Sontheimer 2009). Nonetheless, key differences exist: miRNA can bind partially to multiple mRNA targets, enabling broad regulatory functions, whereas siRNAs exhibit high specificity, binding exclusively to a single complementary mRNA, thus offering distinct clinical applications (Lam et al. 2015).

Therapeutic application of miRNAs can be categorized into miRNA inhibition and miRNA replacement (Lam et al. 2015). Certain miRNAs have been implicated in AD pathology, with elevated levels detected in the brains of AD patients, including miR‐125b, miR‐146a, miR‐155, miR‐101, miR‐106, and miR‐423 (Delay et al. 2012; Wang et al. 2014; Reddy et al. 2017; Zhao et al. 2020). Consequently, miRNA inhibition represents a potential strategy to suppress their pathological effects.

Conversely, miRNA replacement therapy has emerged as an alternative strategy to restore the expression of downregulated miRNAs in AD, such as miR‐29, miR‐107, and miR‐181c (Delay et al. 2012; Wang et al. 2014), and to enhance the levels of neuroprotective miRNAs. For instance, overexpressing miRNAs related to neuronal processes, including neuron differentiation, for example, miR‐135, miR‐153, and miR‐183 (Wang et al. 2014), and thereby counteracting hippocampal neurogenesis deficits linked to cognitive impairment in AD (Farioli‐Vecchioli et al. 2022; Geigenmüller et al. 2024) represents an interesting approach. Notably, a study involving AD patients undergoing a three‐month aerobic exercise regimen found that serum miR‐129‐5p levels were upregulated and positively correlated with Mini‐Mental State Examination (MMSE) score (Li, Xie, et al. 2020), a cognitive assessment scale used in the investigation of patients at risk of dementia, suggesting a promising role for miRNA replacement strategy.

Given that EV‐mediated miRNA delivery is a naturally occurring mechanism extensively studied in both physiological and pathological contexts (Melo et al. 2014; Lee et al. 2019; Gayen et al. 2020; Li et al. 2025), it has emerged as a potential therapeutic approach. Enriching EVs with specific miRNAs to target pathological cells holds promise for treating various diseases, particularly neurodegenerative disorders. In an intracerebral hemorrhage rat model, miR‐133b‐enriched EVs administered intravenously activated neuroprotective and anti‐apoptotic pathways (Shen et al. 2018) (Table 1). Furthermore, EV‐mediated miRNA communication was also evidenced in a study comparing treatment with EVs obtained from adipose‐derived mesenchymal stem cells (ADMSCs) and EVs derived from ADMSCs pre‐treated with an anti‐miR‐25‐3p oligonucleotide (Kuang et al. 2020). In the research, it was demonstrated that EVs derived from ADMSCs led to enhanced neurological recovery in mice subjected to cerebral ischemia, an effect attenuated when EVs were pre‐treated with an anti‐miR‐25‐3p oligonucleotide (Kuang et al. 2020) (Table 1). Collectively, these findings highlight the potential of miRNA‐enriched EVs as a therapeutic strategy for AD (Figure 1).

Additionally, siRNA‐based therapies offer a highly specific gene‐silencing approach. It is a gene knockdown‐based method that functions through sequence‐specific binding to mRNA. This interaction leads to mRNA degradation, inhibition of gene expression, and therefore, reduction in related protein (Alshaer et al. 2021; Friedrich and Aigner 2022). This mechanism makes siRNAs particularly suitable for silencing disease‐related genes. In AD, siRNA‐based therapies have targeted tau or Aβ dysfunction. Accordingly, studies have demonstrated that BACE1‐targeting siRNAs can reduce Aβ accumulation and improve cognitive function (Zhou et al. 2020; Yang et al. 2022). In a different approach, Shin et al. targeted a cell cycle regulator associated with aging with siRNA to rejuvenate aged microglia, as it has less ability to phagocytose amyloid. The treatment increased Aβ clearance and mitigated cognitive decline in an AD mouse model (Shin et al. 2024). Similarly, genes associated with an elevated AD risk have been explored using siRNA as a therapeutic strategy. Intracerebroventricular administration of brain apolipoprotein E (APOE)‐target siRNA has been shown to reduce amyloid plaque formation in a transgenic mouse model of AD (Ferguson et al. 2024).

Despite its therapeutic promise, siRNA therapy faces challenges related to instability, elimination, and off‐target effects (Hu et al. 2020; Alshaer et al. 2021). To address these limitations, protecting siRNA's function and maximizing efficacy, encapsulation within nanocarriers, including EVs, has been proposed. EVs offer a promising delivery system due to their small size, lipid composition, and low immunogenicity. Several strategies for siRNA encapsulation in EVs have been explored, such as calcium phosphate nanoparticles, cholesterol‐conjugated siRNAs, and transient membrane disruption through sonication and electroporation (Alvarez‐Erviti et al. 2011; Lamichhane et al. 2016; O'Loughlin et al. 2017; Roerig et al. 2022).

Corroborating the periphery‐to‐brain communication capacity of EVs, Alvarez‐Erviti et al. demonstrated that the fusion of exosomes with rabies virus glycoprotein peptide (RVG), a rabies virus glycoprotein peptide, loaded with BACE1 siRNA and intravenously injected into wild‐type mice silenced BACE1 mRNA and protein levels by 60% and 62%, respectively (Alvarez‐Erviti et al. 2011) (Table 1). In a distinct strategy, Li et al. designed lesion‐recognizing nanoparticles containing RVG peptide‐fused mesenchymal stem cells (MSCs)‐derived exosomes loaded with BACE1 and caspase‐3 siRNA to treat transgenic AD mice. The exosomes were injected intranasally and efficiently reached the brain and fused with the neuronal membranes, resulting in downregulation of BACE1 and caspase‐3, a reduction in the number of reactive astrocytes and Aβ plaques, restoration of neuronal integrity, and alleviation of cognitive deficits (Li et al. 2023) (Table 1). These studies highlight the promising advantages of siRNA‐loaded EV‐based AD therapy (Figure 1).

Although miRNA‐ and siRNA‐enriched EVs raised interest as a potential therapeutic approach, some challenges remain to be addressed so that they can be tested in clinical trials. Firstly, the therapeutic effects depend on the quantity of these short RNAs. Although different methods exist to encapsulate miRNA and siRNA into EVs, the low efficiency is still a barrier to overcome (Gorshkov et al. 2022). For instance, in the case of siRNA, electroporation is the primary method and achieves only a 20% to 30% success rate, indicating the need for a large quantity of EVs (Li et al. 2024).

However, large‐scale production of EVs also presents a challenge. miRNA‐ and siRNA‐enriched EVs are typically developed through transfection in MSCs or adipose tissue‐derived stem cells (AdSCs) (Munir et al. 2020; Li et al. 2024). Nevertheless, the secretion rates of EVs from cell cultures remain insufficient (Amiri et al. 2022). Although efforts have been made to overcome this limitation through the production of extracellular vesicle mimetics (Fu et al. 2021), further studies are required.

Lastly, as EV research is still emerging, the isolation and purification of EVs remain a major challenge. The most used isolation methods, including ultracentrifugation, immunoaffinity‐based techniques, and size‐exclusion chromatography, are costly and have limited purification efficiency due to the co‐precipitation of non‐EV contaminants with overlapping sizes (Amiri et al. 2022; Gorshkov et al. 2022). Therefore, the development of more standardized and optimized methods, along with the establishment of good manufacturing practice guidelines, is essential for clinical application.

### 6.3 Immunotherapy

Immunotherapy represents an advanced strategy in the context of neurodegenerative diseases. This approach uses the immune system or its derivative components to enhance the existing CNS immune response and facilitate the clearance of aggregated toxic proteins. Specifically in AD, both active and passive immunotherapy strategies have been investigated in clinical and preclinical models, primarily focused on targeting inflammatory agents to mitigate neuroinflammation and ameliorate AD symptoms (Heneka et al. 2013; Potter et al. 2021; Ou et al. 2022; Gerhard et al. 2023).

Recent advances in immunotherapy have led to the proposal of monoclonal antibodies as a strategy for clearing pathological factors, such as Aβ and Tau aggregates (Song et al. 2022). The most promising treatment developments targeting Aβ protein in AD are Lecanemab and Donanemab (Rafii and Aisen 2025). In a phase III trial, biweekly intravenous administration of Lecanemab, a monoclonal antibody specifically targeting soluble Aβ protofibrils, reduced Aβ burden and slowed clinical progression in early AD over an 18‐month period (Van Dyck et al. 2023). Similarly, in a distinct phase III trial, Donanemab, which targets pyroglutamate‐modified Aβ plaques, cleared Aβ aggregation and slowed clinical progression in early AD with both amyloid and tau pathology after once‐monthly intravenous administration for 19 months (Sims et al. 2023). Both Lecanemab and Donanemab have recently received FDA approval for the treatment of early AD (Heneka, Morgan, et al. 2024). Conversely, several phase II clinical trials have investigated the use of anti‐tau antibodies; however, no significant effects have been observed in slowing AD progression (Teng et al. 2022; Florian et al. 2023; Shulman et al. 2023).

Despite significant advances, monoclonal antibody‐based therapies for AD continue to face limitations in their therapeutic effectiveness. A primary challenge to these therapies arises from the antibody's ability to cross the BBB and bind to the intended target. The highly selective nature of the BBB, along with the potential for adverse effects associated with monoclonal antibody treatments, represents major obstacles to clinical success (Song et al. 2022). Consequently, alternative delivery strategies have been explored to overcome these challenges and enhance antibody targeting, thereby improving the overall efficacy of immunotherapy.

The utilization of EVs or nanoparticle‐based delivery systems has been explored as a strategy to potentiate immunotherapy's efficacy. These systems aim to elevate specificity and safety while also capitalizing on EVs' inherent ability to cross the BBB. EV‐based immunotherapy was first proposed in 1998 by Zitvogel and collaborators (Zitvogel et al. 1998), who evaluated the therapeutic effects of dendritic cell‐derived EVs in tumor‐bearing mice. In recent years, extensive research in oncology has been conducted to investigate the potential benefits of EV‐based immunotherapy. For instance, engineered dendritic cell‐derived EVs, modified with ovalbumin, LPS, and interferon‐gamma (IFN‐γ), were shown to induce immunostimulatory responses in macrophages, T cells, and dendritic cells in tumor‐bearing mice (Matsumoto et al. 2020). In a different approach, the overexpression of interleukin‐12 (IL‐12) and/or the suppression of transforming growth factor‐beta (TGF‐β) increased the antitumor immune response in a preclinical colon carcinoma model (Rossowska et al. 2019).

Although EV‐based immunotherapy is not yet an extensively explored topic within the AD field, some studies have proposed the application of nanoparticles as delivery systems in preclinical AD models. In a transgenic mouse model of AD, intravenous administration of a polymeric nanomicelle system successfully delivered antibody fragments capable of binding to Aβ in the brain. This resulted in increased accumulation of the antibody fragments in the brain compared to the administration of the antibody fragments alone, contributing to elevated therapeutic effects (Xie et al. 2020). Notably, this delivery system also demonstrated minimal uptake in peripheral tissues (Xie et al. 2020). In an alternative strategy, treatment with a zwitterionic poly(carboxybetaine) (PCB)‐based nanoparticle specifically targeted microglia, leading to optimized Aβ phagocytosis and attenuated memory impairment in a transgenic AD mouse model (Liu et al. 2020).

EVs represent an innovative delivery method that can address some inherent limitations of immunotherapy, particularly due to their ability to cross the BBB and to transport bioactive molecules. EVs also present other favorable characteristics, including biocompatibility, safety, stability, and targeting specificity. Therefore, EVs demonstrate significant potential as a delivery system for immunotherapeutic approaches in the treatment of AD.

### 6.4 Clinical Trials

Considering the promising therapeutic role of EVs and the anti‐inflammatory properties of MSCs, EVs‐MSCs have attracted attention as a potential treatment approach. EV‐MSCs represent a cell‐free therapy with lower immunogenicity and no risk of tumor formation (Yin et al. 2019; Herrmann et al. 2021). Consequently, ongoing clinical trials are investigating this technique for various diseases, including infectious diseases, autoimmune disorders, and neurological disorders (e.g., NCT04270006, NCT04544215, NCT04491240, NCT06813027, NCT03384433). Notably, a clinical trial utilizing siRNA‐loaded EVs‐MSCs is currently underway for the treatment of metastatic pancreatic cancer (e.g., NCT03608631), underscoring the potential of EVs as an siRNA delivery system. Furthermore, a clinical trial is evaluating the safety and efficacy of allogeneic EVs‐ADMSC in patients with AD (e.g., NCT04388982).

Extensive research, as previously mentioned, has highlighted the potential of EVs as advanced drug delivery systems. EVs offer several advantages, primarily due to their endogenous origin, low immunogenicity, and ability to cross the BBB—a crucial characteristic for prospective AD therapies. Currently, diverse approaches for incorporating bioactive molecules within EVs have been studied, including exercise‐induced and anti‐inflammatory molecules, miRNAs, and siRNAs (Table 1). Despite existing challenges, such as optimizing isolation methodologies, refining purification protocols, and enhancing encapsulation efficiency, EV‐based therapeutics hold significant promise as a novel approach for AD treatment.

## 7 Conclusions

In conclusion, EVs hold a pivotal component for elucidating intercellular communication networks in the healthy brain and in AD pathophysiology. EVs can facilitate the propagation of misfolded proteins related to AD, thereby contributing to the advance of the disease. In contrast, due to their ability to cross the BBB and serve as mirrors of the CNS, EVs can act as early indicators for AD. Importantly, EVs can be isolated from accessible biofluids, such as blood or saliva, offering a minimally invasive diagnostic tool.

Despite ongoing research efforts, therapeutic options for AD remain limited. Current treatments primarily target disruptions in neurotransmitter signaling or aim to mitigate pathological protein accumulation, yielding only modest symptomatic relief and minimal impact on the overall progression of the disease (Sarazin et al. 2024). Interestingly, EVs are being considered as drug transporters. Their small size, low immunogenicity, and natural capacity to cross the BBB make them promising candidates as vehicles for medications. Hence, the emerging field of EVs represents a promising advance for potential AD treatments, particularly given the ongoing lack of a treatment capable of reversing cognitive decline in affected individuals.

Although there are challenges, such as standardization of isolation methods and full understanding of their biodistribution, the use of EVs as diagnostic and therapeutic tools represents a rapidly evolving and promising field. Therefore, continued research is essential to explore the multifaceted roles of EVs in AD.

## Author Contributions

**Anna R. R. Da Conceicao:** conceptualization, investigation, writing – original draft, methodology, validation, visualization, writing – review and editing. **Júlia Marinatto:** conceptualization, investigation, writing – original draft, methodology, validation, visualization, writing – review and editing. **Lisandra S. Pinheiro:** conceptualization, investigation, writing – original draft, methodology, validation, visualization, writing – review and editing. **Tayna Rody:** conceptualization, investigation, writing – original draft, methodology, validation, visualization, writing – review and editing. **Fernanda G. De Felice:** conceptualization, investigation, funding acquisition, writing – original draft, methodology, validation, visualization, writing – review and editing, project administration, supervision, resources.

## Ethics Statement

All experiments were conducted in compliance with the ARRIVE guidelines.

## Consent

Informed consent was achieved for all subjects, and the experiments were approved by the local ethics committee.

## Conflicts of Interest

The authors declare no conflicts of interest.

## Peer Review

The peer review history for this article is available at https://www.webofscience.com/api/gateway/wos/peer‐review/10.1111/jnc.70209.

## Data Availability

Data sharing not applicable to this article as no datasets were generated or analysed during the current study.
